# Fusion Protein Vaccines Targeting Two Tumor Antigens Generate Synergistic Anti-Tumor Effects

**DOI:** 10.1371/journal.pone.0071216

**Published:** 2013-09-13

**Authors:** Wen-Fang Cheng, Ming-Cheng Chang, Wei-Zen Sun, Yu-Wei Jen, Chao-Wei Liao, Yun-Yuan Chen, Chi-An Chen

**Affiliations:** 1 Departments of Obstetrics and Gynecology, College of Medicine, National Taiwan University, Taipei, Taiwan; 2 Graduate Institute of Oncology, College of Medicine, National Taiwan University, Taipei, Taiwan; 3 Graduate Institute of Clinical Medicine, College of Medicine, National Taiwan University, Taipei, Taiwan; 4 Department of Anesthesiology, College of Medicine, National Taiwan University, Taipei, Taiwan; 5 Animal Technology Institute Taiwan, Miaoli, Taiwan; Federal University of São Paulo, Brazil

## Abstract

**Introduction:**

Human papillomavirus (HPV) has been consistently implicated in causing several kinds of malignancies, and two HPV oncogenes, E6 and E7, represent two potential target antigens for cancer vaccines. We developed two fusion protein vaccines, PE(ΔIII)/E6 and PE(ΔIII)/E7 by targeting these two tumor antigens to test whether a combination of two fusion proteins can generate more potent anti-tumor effects than a single fusion protein.

**Materials and Methods:**

*In* *vivo* antitumor effects including preventive, therapeutic, and antibody depletion experiments were performed. *In* *vitro* assays including intracellular cytokine staining and ELISA for Ab responses were also performed.

**Results:**

PE(ΔIII)/E6+PE(ΔIII)/E7 generated both stronger E6 and E7-specific immunity. Only 60% of the tumor protective effect was observed in the PE(ΔIII)/E6 group compared to 100% in the PE(ΔIII)/E7 and PE(ΔIII)/E6+PE(ΔIII)/E7 groups. Mice vaccinated with the PE(ΔIII)/E6+PE(ΔIII)/E7 fusion proteins had a smaller subcutaneous tumor size than those vaccinated with PE(ΔIII)/E6 or PE(ΔIII)/E7 fusion proteins alone.

**Conclusion:**

Fusion protein vaccines targeting both E6 and E7 tumor antigens generated more potent immunotherapeutic effects than E6 or E7 tumor antigens alone. This novel strategy of targeting two tumor antigens together can promote the development of cancer vaccines and immunotherapy in HPV-related malignancies.

## Introduction

Cervical cancer is the second leading cause of cancer death in women, with approximately 500,000 cases worldwide each year, of which about one-third are fatal [1]. Human papillomavirus (HPV) is recognized as the primary cause of cervical cancer as HPV DNA can be detected in about 99% of all cervical cancers [2]. HPV 16, in particular, is the most prevalent type and is detected in over 50% of patients [3]. The HPV types found in cancer cells have been shown to have the ability to transform in *in vitro* studies [4], and the viral transforming proteins, E6 and E7, have consistently been shown to be expressed in cervical cancer cell lines [5] and in HPV-associated cancers [6]. In HPV-associated malignant transformation, viral DNA may be integrated into the cellular DNA, often resulting in the deletion of large sectors of the viral genome. Expressions of E6 and E7 are likely to overcome the regulation of cell proliferation normally mediated by proteins such as p53 and Rb, allowing for uncontrolled growth and providing the potential for malignant transformation [7]. Thus, E6 and E7 are two oncogenic proteins that represent ideal target antigens for developing vaccines and immunotherapeutic strategies against HPV-associated neoplasia.

Pre-clinical and clinical studies have targeted E6 or E7 for the development of vaccines to control HPV-associated lesions [8]. Most HPV researchers have focused on E7 such that E7 as the immuno-dominant epitope and the associated immune responses have been well characterized [9,10]. Since E6 represents another important target for vaccines to control HPV-associated lesions, it is crucial to develop vaccines targeting E6.

Ideal cancer treatment should be able to eradicate systemic tumors at multiple sites in the body while having the specificity to discriminate between neoplastic and non-neoplastic cells. As such, the activation of antigen-specific T cell-mediated immune responses allows for the killing of tumors associated with a specific antigen [10,11]. Recently, many strategies such as peptide-based vaccine [12,13], protein-based vaccine [14,15], DNA-based vaccine [16,17], naked RNA vaccine [18,19], and recombinant viruses [20,21] have emerged. Protein-based vaccines are reportedly capable of generating CD8^+^ T cell responses in vaccinated humans [22,23]. However, one limitation of peptide and protein-based vaccines is their poor immunogenicity, especially with some tumor antigens such as E6 and E7. Novel strategies that enhance protein vaccine potency need to be applied in the development of more effective cancer vaccines and immunotherapy.

A previous study revealed that a fusion protein vaccine with the translocation features of exotoxin A of *Pseudomonas aeruginosa* (PE(ΔIII)-KDEL3) linked with the model antigen-human papillomavirus (HPV) 16 E7 enhanced the MHC class I presentation of antigens to CD8^+^ T cells and thereby enhanced vaccine potency [15]. Another previous study revealed that a DNA vaccine encoding HSP60 linked to E6 and/or E7 generated significantly enhanced E6 or E7-specific CD8^+^ T cell responses in vaccinated mice, and prevented and controlled lethal E6 and E7-expressing tumors [24]. The chimeric HSP60/E6/E7 DNA vaccine also generated more potent immunotherapeutic effects than the chimeric HSP60/E6 or HSP60/E7 DNA vaccines. However, few studies have focused on E6 as the target antigen [25]. The present study aimed to determine whether retrograde-delivery domains, when linked to the E6 antigen in the fusion protein format, could also enhance E6-specific immunologic responses and anti-tumor effects, and whether the combination of E6 and E7 fusion protein vaccines could generate a more potent antigen-specific immunotherapy than E6 or E7 fusion protein vaccines alone.

## Materials and Methods

### Preparation of various DNA constructs

The E6, E7, PE(ΔIII)/E6, and PE(III)/E7 preparations were done as described previously with some modifications [15,24]. Briefly, the wild-type E7 construct was removed and then the wild-type E6 construct was inserted.

### Generation and Preparation of Various Protein Vaccines

The induction of expression, production, and purification of various recombinant proteins were also done as described previously [15]. After purification, protein elution fractions were analyzed for purity and quantified by SDS-PAGE analysis.

### Preparation and vaccination of protein vaccines

Stock protein vaccines such as E6, E7, PE(ΔIII)/E6, and PE(ΔIII)/E7 were diluted with PBS (1:10) 10 times and incubated for 2 hours at 37°C. The activated proteins were further mixed with 10% ISA206 (SEPPIC Inc., Paris, France) for the *in vitro* and *in vivo* experiments. For the *in vivo* experiments, various ISA206 mixed protein vaccines were subcutaneously injected into the back of the study mice.

### Cell lines

The production and maintenance of TC-1 tumor cells, E6- and E7-specific CD8^+^ T cell lines, were done as previously described [26,27]. On the day of tumor challenge, tumor cells were harvested by trypsinization, washed twice with 1X Hanks buffered salt solution (HBSS) and re-suspended in 1X HBSS to the designated concentration for injection.

### Mice

Six- to eight-week-old female C57BL/6J mice were purchased from the National Taiwan University (Taipei, Taiwan) and bred in the animal facility of the National Taiwan University. The Institutional Animal Care and Use Committee of National Taiwan University approved this study. All animal procedures were performed according to approved protocols and strictly adhered to the guidelines set by the committee for the proper use and care of laboratory animals.

### Intracellular cytokine staining and flow cytometry analysis

For the first experiment, mice (5 per group) were immunized with a total of 0.1 mg of various protein vaccines mixed with 10% ISA206 adjuvant. The mice were then boosted subcutaneously one and two weeks later with the same regimen. One week after the last immunization, the mice were sacrificed and splenocytes were prepared as described previously [28]. To detect E6-specific immunologic responses, E6-specific peptide (aa 50-57) containing an MHC class I epitope [25] to detect E6-specific CD8^+^ T cell precursors or 50 µg/ml E6 recombinant protein [15] to detect E6-specific CD4^+^ T cell precursors were used. To detect E7-specific immunological responses, E7-specific peptide (aa 49-57) containing an MHC class I epitope [29] to detect E7-specific CD8^+^ T cell precursors or 10 µg/ml E7 peptide (aa 30-67) containing an MHC class II epitope [17] to detect E7-specific CD4^+^ T cell precursors were used.

Before intracellular cytokine staining, 3.5x10^5^ pooled splenocytes from each vaccinated group were incubated for 16 hours with either peptide (or recombinant protein) to detect E6- or E7-specific CD4^+^ or CD8^+^ T cell precursors. Cell surface marker staining for CD8 or CD4, and intracellular cytokine staining for IFN-γ, as well as FACScan analysis were performed [16].

In the second experiment, the mice were immunized at various times with PE(ΔIII)/E6, PE(ΔIII)/E7, or PE(ΔIII)/E6+PE(ΔIII)/E7 fusion proteins as described earlier. The mice were sacrificed 7 days after the last vaccination. Splenocytes were harvested, prepared, stained, and analyzed.

### Enzyme-linked immuno-absorbent assay (ELISA) for anti-E6 or E7 antibodies

Mice (5 per group) were vaccinated with various protein vaccines three times as described earlier. Sera were prepared from the mice 14 days after the last immunization. E6- or E7-specific antibody titers were detected by direct ELISA as previously described [17,30].

### *In vivo* tumor protection experiments

For the first experiment, mice (5 per group) were subcutaneously vaccinated with a total of 0.1 mg of various protein vaccines and boosted one or two weeks later with the same regimen. One week after the last vaccination, the mice were challenged with 5x10^4^ TC-1 tumor cells by subcutaneous injection in the right leg. Naive mice received the same amount of TC-1 cells to assess natural tumor growth and served as the controls. Tumor growth was monitored by visual inspection and palpation twice weekly until 60 days after tumor challenge. Tumor-free mice were defined as those with no grossly visible or palpable tumor nodules.

For the second experiment, mice (5 per group) were vaccinated with 0.1 mg PE(ΔIII)/E6, PE(ΔIII)/E7, or PE(ΔIII)/E6+PE(ΔIII)/E7 fusion proteins once or twice. One week after the last vaccination, the mice were challenged with 5x10^4^ TC-1 tumor cells subcutaneously and tumor growth was monitored.

### *In vivo* Ab depletion experiments

*In vivo* Ab depletion experiments were performed as described previously with some modifications [16,17]. Mice (5 per group) were first injected intraperitoneally with the respective Abs on day 0, challenged with 5xl0^4^ cells/mouse TC-1 tumor cells via intravenous tail vein injection on day 3, and immunized subcutaneously with 0.1 mg PE(ΔIII)/E6, 0.1 mg PE(ΔIII)/E7, or 0.05 mg PE(ΔIII)/E6 + 0.05 mg PE(ΔIII)/E7 protein vaccines boosted one and two weeks after day 7 to determine the effects of lymphocyte subsets on the potency of the PE(ΔIII)/E6 and/or PE(ΔIII)/E7 protein vaccines. The mAb GK1.5 was used for CD4 depletion, mAb 2.43 for CD8 depletion, and mAb PK136 for NK1.1 depletion. Depletion was terminated on the day of sacrifice 28 days after TC-1 tumor challenge.

### *In vivo* tumor treatment experiments

*In vivo* tumor treatment experiments were performed using a previously described lung hematogenous spread model [16,18]. In the first experiments, mice (5 per group) were challenged with 5xl0^4^ TC-1 tumor cells/mouse via tail vein injection. Three or seven days after tumor challenge, the mice received a total of 0.1 mg/mouse of various protein vaccines subcutaneously every 7 days for 2 weeks (total of 0.3 mg protein). The mice that were not vaccinated were used as negative controls. The mice were sacrificed and their lungs were explanted on day 30. Pulmonary tumor nodules in each mouse were evaluated and counted by investigators blinded to the sample identity.

The second experiments were the subcutaneous therapeutic experiments. C57BL/6 mice (5 per group) were challenged with 5x10^4^ TC-1 tumor cells/mouse subcutaneously on the right leg to generate subcutaneous tumor nodules in the morning of day 0. In the morning of day 3 after tumor challenge, the mice received various protein vaccines and every week thereafter. Perpendicular tumor diameters were measured using Vernier scale calipers, while tumor volume was calculated using the formula [(*x* ^2^ *y*)/2] for an ellipsoid. The mice were monitored twice a week and sacrificed if the tumor volume became > 1,200 mm^3^.

### Statistical analysis

All data were expressed as mean±SEM. Experimental groups were compared using analysis of variance (ANOVA) using the Statistical Package for Social Sciences (SPSS) software (SPSS 9.0, SPSS Inc., Chicago, IL). Statistical significance was set at *p*<0.05.

## Results

### Generation and characterization of the E6 and PE(ΔIII)/E6 protein vaccines

**Figure 1:**
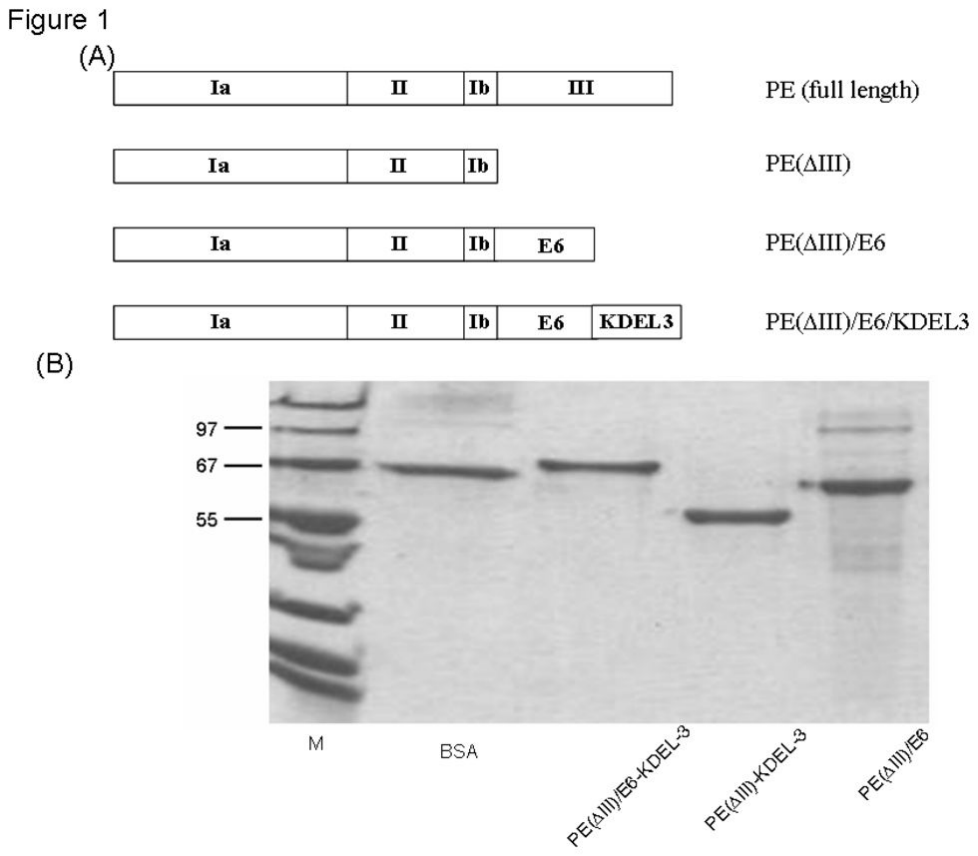
Chimeric PE(ΔIII)/E6 DNA construct and characterization of PE(ΔIII)/E6 fusion protein expression. (**A**) The schematic diagram of the constructs for the full-length exotoxin and PE(ΔIII), PE(ΔIII)-KDEL-3, and PE(ΔIII)-E6-KDEL-3 genes. (**B**) The SDS-PAGE pattern of various E6 fusion proteins. Lane 1, BSA standard; Lane 2, PE(ΔIII)-E6-K3; Lane 3 PE(ΔIII)-K3,; Lane 4, PE(ΔIII)-E6.

The generation and characterization of the PE(ΔIII)/E7 protein were done as described in a previous study [15]. A schematic diagram showing the domains of full-length PE and the construct of E6, and chimeric PE(ΔIII)**/**E6 is shown in Figure **1A**, and the SDS-PAGE findings of PE(ΔIII), E6, and PE(ΔIII)**/**E6 are shown in Figure **1B**.

### Combination of PE(ΔIII)/E6 and PE(ΔIII)/E7 fusion proteins generated potent E6- and E7-specific immune responses *in vivo*

**Figure 2:**
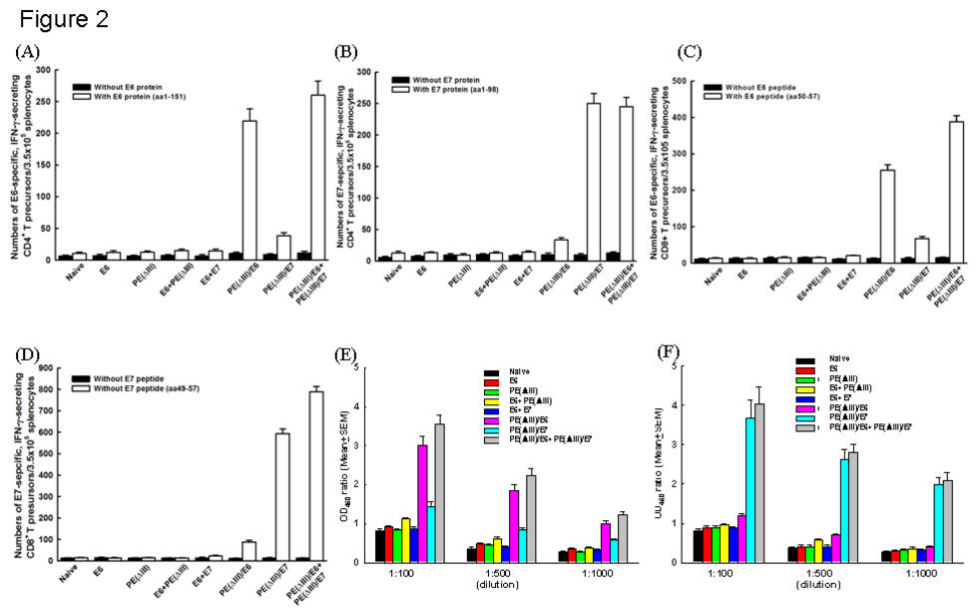
Immunologic profiles of the vaccinated mice using intracellular cytokine staining and ELISA. (**A**) The number of IFN-γ-secreting CD4^+^ T cells with (open columns) or without (filled columns) the corresponding E6 protein (aa1-151). *Note*: The numbers of E6-specific, IFN-γ-secreting CD4^+^ T cells in the PE(ΔIII)/E6 (219.5±18.9) and PE(ΔIII)/E6+PE(ΔIII)/E7 (260.0±22.4) groups were significantly higher than those in the other groups (*p*<0.01, one-way ANOVA). (**B**) The number of IFN-γ-secreting CD4^+^ T cells in with (open columns) or without (filled columns) the corresponding E7 protein (aa1-98). *Note*: The numbers of E7-specific, IFN-γ-secreting CD4^+^ T cells in the PE(ΔIII)/E7 (258.0±18.2) and PE(ΔIII)/E6+PE(ΔIII)/E7 (239.5±17.5) groups were significantly higher than those in the other groups (*p*<0.01, one-way ANOVA). (**C**) The number of IFN-γ-secreting CD8^+^ T cell precursors with (open columns) or without (filled columns) the corresponding E6 peptide (aa 50-57). *Note*: The PE(ΔIII)/E6 (247.5±18.2) and PE(ΔIII)/E6+PE(ΔIII)/E7 (392.5±19.6) groups generated higher numbers of E6-specific, IFN-γ-secreting CD8^+^ T precursors than the other groups (*p*<0.01, one-way ANOVA). (**D**) The number of IFN-γ-secreting CD8^+^ T cell precursors with (open columns) or without (filled columns) the corresponding E7 peptide (aa 49-57). *Note*: The PE(ΔIII)/E7 (588.5±16.8) and PE(ΔIII)/E6+PE(ΔIII)/E7 (778.0±18.9) groups also generated higher numbers of E7-specific, IFN-γ-secreting CD8^+^ T precursors than the other groups (*p*<0.01, one-way ANOVA). ELISA demonstrated (**E**) E6-specific and (**F**) E7-specific Abs in the mice vaccinated with various protein vaccines. *Note*: The titers of E6-specific Abs were higher in the PE(ΔIII)/E6 and PE(ΔIII)/E6+PE(ΔIII)/E7 groups than in the other groups (*p*<0.01, one-way ANOVA). The PE(ΔIII)/E7 and PE(ΔIII)/E6+PE(ΔIII)/E7 also generated significantly higher titers of E7-specific Abs than the other groups (*p*<0.01, one-way ANOVA).

The numbers of E6-specific, IFN-γ-secreting CD4^+^ T cells in the PE(ΔIII)/E6 (219.5±18.9) and PE(ΔIII)/E6+PE(ΔIII)/E7 (260.0±22.4) groups were significantly higher than those in the E6 (12.0±2.8), E6+E7 (14.5±2.1), and PE(ΔIII)/E7 (38.5±4.9) groups (*p*<0.01, one-way ANOVA) (Figure **2A**). Similarly, the numbers of E7-specific, IFN-γ-secreting CD4^+^ T cells in the PE(ΔIII)/E7 (258.0±18.2) and PE(ΔIII)/E6+PE(ΔIII)/E7 (239.5±17.5) groups were significantly higher than those in the E6 (15.0±2.8), E6+E7 (18.5±2.1), and PE(ΔIII)/E7 (77.5±7.0) groups (*p*<0.01, one-way ANOVA) (Figure **2B**).

The PE(ΔIII)/E6 (247.5±18.2) and PE(ΔIII)/E6+PE(ΔIII)/E7 (392.5±19.6) groups generated higher numbers of E6-specific, IFN-γ-secreting CD8^+^ T precursors than the E7 (14.5±2.1), E6+E7 (18.0±2.8), and PE(ΔIII)/E7 (33.5±2.8) groups (*p*<0.01, one-way ANOVA) (Figure **2C**). The PE(ΔIII)/E7 (588.5±16.8) and PE(ΔIII)/E6+PE(ΔIII)/E7 (778.0±18.9) groups also generated higher numbers of E7-specific, IFN-γ-secreting CD8^+^ T precursors than the E7 (12,5±2.1), E6+E7 (16.5±2.8), and PE(ΔIII)/E6 (89.0±7.7) groups (*p*<0.01, one-way ANOVA) (Figure **2D**).

The titers of E6-specific Abs were higher in the PE(ΔIII)/E6 (3.010±0.180 1: 100 dilution) and PE(ΔIII)/E6+PE(ΔIII)/E7 (3.610±0.195 1: 100 dilution) groups than those in the other groups (Figure **2E**) (*p*<0.01, one-way ANOVA). The PE(ΔIII)/E7 (3.690±0.215 1: 100 dilution) and PE(ΔIII)/E6+PE(ΔIII)/E7 (4.040±0.215 1: 100 dilution) groups also generated significantly higher titers of E7-specific Abs than the other groups (Figure **2F**) (*p*<0.01, one-way ANOVA).

Thus, the PE(ΔIII)/E6 and PE(ΔIII)/E7 proteins generated E6- and E7-specific potent immune responses, respectively. Moreover, PE(ΔIII)/E6+PE(ΔIII)/E7 generated both E6- and E7-specific potent immune responses.

### Vaccination with PE(ΔIII)/E6 and/or PE(ΔIII)-E7 fusion proteins generated potent tumor protection in mice challenged with E6- and E7-expressing tumor cells

**Figure 3:**
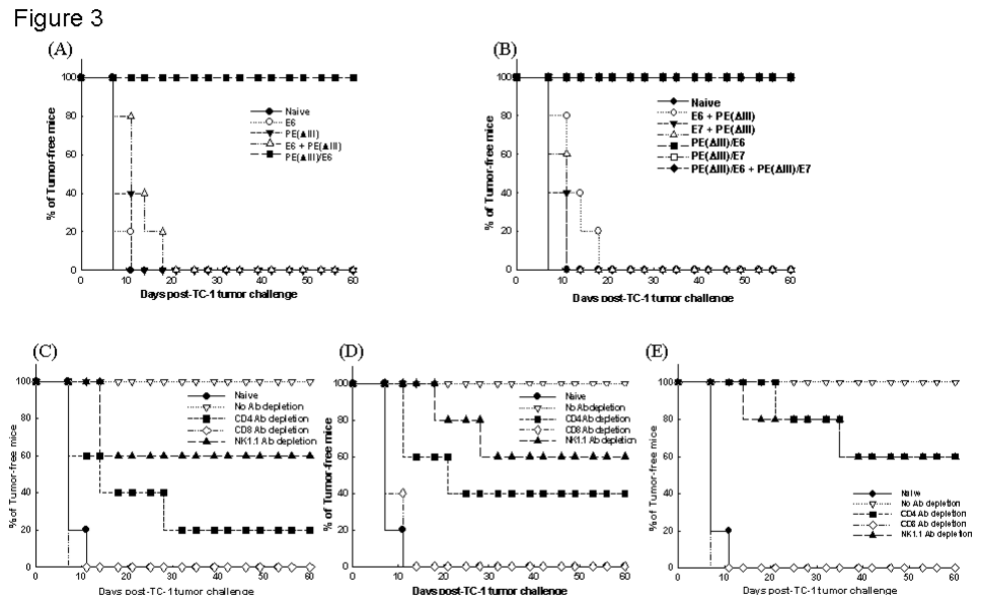
*In vivo* tumor prevention and Ab depletion experiments in mice vaccinated with PE(ΔIII)/E6 and/or PE(ΔIII)/E7 protein vaccines. *In* *vivo* tumor protection experiments of the mice vaccinated with (**A**) respective E6 protein vaccines and (**B**) E6 and/or E7 fusion protein vaccines. *In* *vivo* Ab depletion experiments of mice vaccinated with (**C**) PE(ΔIII)/E6 protein vaccine, (**D**) PE(ΔIII)/E7 vaccine, and (**E**) PE(ΔIII)/E6+PE(ΔIII)/E7 protein vaccines.

In the *in vivo* tumor protection experiments, 100% of the mice that received the PE(ΔIII)/E6 fusion protein vaccine remained tumor-free for 60 days after TC-1 challenge (Figure **3A**). In contrast, all of the unvaccinated mice and mice receiving E6, PE(ΔIII), PE(ΔIII) +E6 developed tumors within 20 days after tumor challenge. Furthermore, 100% of the mice vaccinated with PE(ΔIII)/E7 or PE(ΔIII)/E6+PE(ΔIII)/E7 fusion protein vaccines were also tumor-free 60 days after TC-1 tumor challenge (Figure **3B**). These results indicated that PE(ΔIII)/E6, PE(ΔIII)/E7, or a combination of PE(ΔIII)/E6 and PE(ΔIII)/E7 fusion proteins generated the same potent tumor protective effects.

### CD4 T cells, CD8 T cells, and NK cells were essential for the anti-tumor effect generated by PE(ΔIII)/E6 and/or PE(ΔIII)/E7 fusion protein vaccines

In the *in vivo* antibody depletion experiments, all naive mice and all PE(ΔIII)/E6 protein-vaccinated mice depleted of CD8^+^ T cells grew tumors within 14 days after tumor challenge (Figure **3C**). In contrast, 80% of the CD4^+^ T cell-depleted mice and 40% of the NK1.1 cell-depleted mice grew tumors within 30 days after tumor challenge. In the PE(ΔIII)/E7 protein-vaccinated mice, 100%, 60%, and 40% of the mice depleted of CD8^+^ T cells, CD4^+^ T cells, and NK1.1 cells, respectively, grew tumors within 30 days after tumor challenge (Figure **3D**), whereas in the mice vaccinated with PE(ΔIII)/E6+PE(ΔIII)/E7 protein vaccines, tumor growth occurred in 100%, 60%, and 60%, respectively (Figure **3E**). Taken together, CD4 T cells, CD8 T cells, and NK cells were essential for the anti-tumor immunity generated by the E6 and E7 fusion protein vaccines.

### Treatment with E6 and/or E7 fusion proteins led to a significant reduction in pulmonary tumor nodules

**Figure 4:**
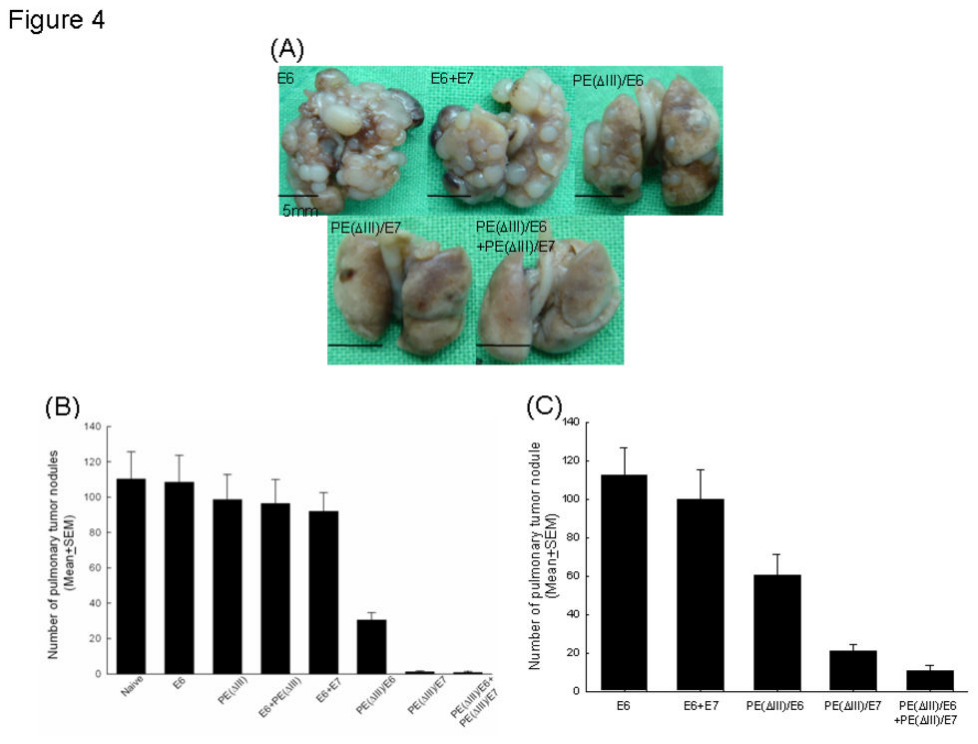
*In vivo* pulmonary metastatic treatment experiments in mice vaccinated with various protein vaccines. (**A**) Representative figures of pulmonary tumor nodules in the treatment experiments of each protein vaccinated group. (**B**) The mean pulmonary tumor nodules in the mice vaccinated with various protein vaccines 3 days after TC-1 tumor injection. *Note*: The mean number of nodules in the mice treated with PE(ΔIII)/E6 (30.6±4.1), PE(ΔIII)/E7 (1.2±0.6), and PE(ΔIII)/E6+PE(ΔIII)/E7 (0.8±0.4) fusion proteins were significantly lower than in the other groups of mice (*p*<0.001, one-way ANOVA). (**C**) The mean pulmonary tumor nodules of the mice treated with various protein vaccines 7 days after TC-1 tumor injection. *Note*: The PE(ΔIII)/E6+PE(ΔIII)/E7 group (10.8±2.5) had the lowest number of pulmonary tumor nodules compared to the PE(ΔIII)/E6 (60.4±10.6) and PE(ΔIII)/E7 (20.8±3.6) groups (*p*<0.01, one-way ANOVA).

The representative figures of pulmonary tumor nodules in various protein vaccinated groups are shown in Figure **4A**. The mean numbers of nodules in the mice treated with PE(ΔIII)/E6 (30.6±4.1), PE(ΔIII)/E7 (1.2±0.6), and PE(ΔIII)/E6+PE(ΔIII)/E7 (0.8±0.4) fusion proteins were significantly lower than those in the mice treated with E6, E7, and E6+E7 proteins, when starting treatment 3 days after TC-1 tumor challenge (*p*<0.001, one-way ANOVA) (Figure **4B**). There was no significant difference in the number of pulmonary tumor nodules between the mice treated with PE(ΔIII)/E7 and those treated with PE(ΔIII)/E6+PE(ΔIII)/E7 (*p*>0.05, one-way ANOVA). However, when starting treatment 7 days after TC-1 tumor challenge, the PE(ΔIII)/E6+E(ΔIII)/E7 group (10.8±2.5) had the lowest number of pulmonary tumor nodules compared to the PE(ΔIII)/E6 (60.4±10.6) and PE(ΔIII)/E7 (20.8±3.6) groups (*p*<0.01, one-way ANOVA) (Figure **4C**). This indicated that both E6 and E7 fusion protein vaccines could control established E7-expressing tumors in the lungs.

### PE(ΔIII)/E6 and PE(ΔIII)/E7 fusion protein vaccines generated E6- and E7-specific T cell immunities and better anti-tumor effects

**Figure 5:**
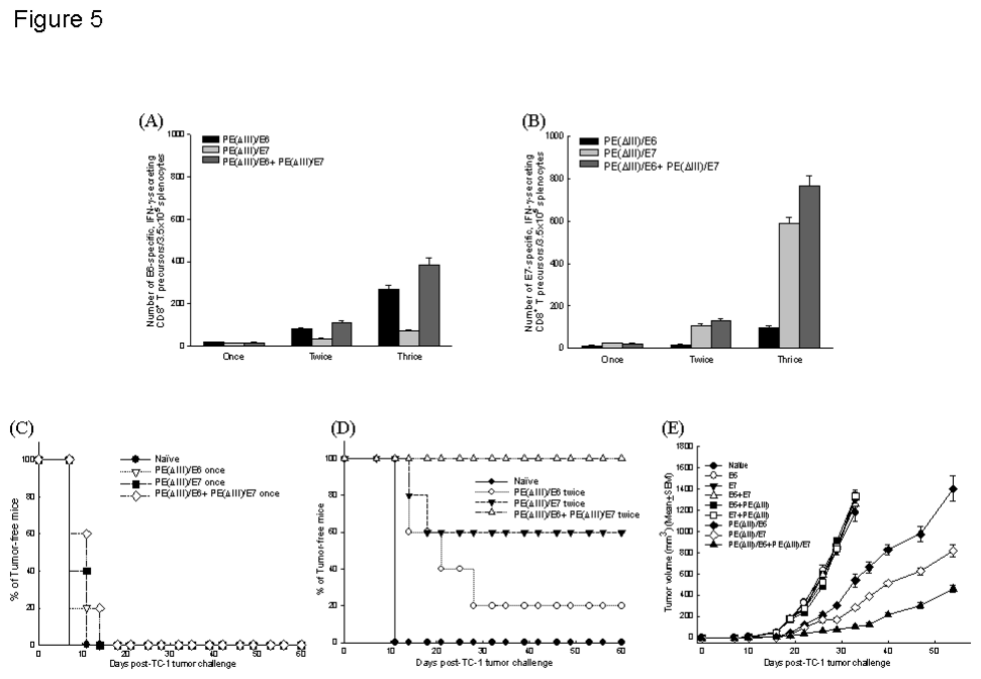
Comparative experiments between mice immunized with various numbers of PE(ΔIII)/E6 and/or PE(ΔIII)/E7 protein vaccines. Numbers of (**A**) E6-specific CD8^+^ T precursors and (**B**) E7-specific CD8^+^ T precursors in the mice vaccinated with varying frequencies of fusion protein vaccines. *Note*: The mice vaccinated more times with PE(ΔIII)/E6+PE(ΔIII)/E7 protein vaccines generated higher numbers of E6- and E7-specific CD8^+^ T precursors. (**C**) Percentages of tumor-free mice in *in* *vivo* prevention experiments of the mice vaccinated once with various fusion protein vaccines. *Note*: All of the naïve mice and mice vaccinated once with PE(ΔIII)/E6 and/or PE(ΔIII)/E7 protein vaccines had tumorigenesis within 14 days after TC-1 tumor challenge. (**D**) Percentages of tumor-free mice in *in* *vivo* prevention experiments of mice vaccinated twice with various fusion protein vaccines. *Note*: 20%, 60%, and 100% of the mice vaccinated twice with PE(ΔIII)/E6, PE(ΔIII)/E7, and PE(ΔIII)/E6+PE(ΔIII)/E7 protein vaccines were tumor-free after 60 days of TC-1 tumor challenge. (**E**) Subcutaneous tumor volumes of the mice in *in* *vivo* therapeutic experiments of mice vaccinated thrice with various fusion protein vaccines. *Note*: The mice vaccinated with the PE(ΔIII)/E6+PE(ΔIII)/E7 fusion protein vaccines had the smallest tumor volumes 55 days after TC-1 tumor challenge (*p*<0.001, one-way ANOVA).

The mice vaccinated with PE(ΔIII)/E6+PE(ΔIII)/E7 protein vaccines had higher numbers of E6-specific CD8^+^ T precursors than those vaccinated with PE(ΔIII)/E6 fusion protein vaccine, especially after three vaccinations (Figure **5A**). The mice vaccinated with PE(ΔIII)/E6+PE(ΔIII)/E7 protein vaccines three times also generated higher numbers of E7-specific CD8^+^ T precursors than those vaccinated with PE(ΔIII)/E7 fusion protein vaccine (Figure **5B**). Moreover, the mice vaccinated more times with PE(ΔIII)/E6+PE(ΔIII)/E7 protein vaccines generated higher numbers of E6- and E7-specific CD8^+^ T precursors (Figure **5A and 5B**).

For the preventive anti-tumor experiments, all of the naïve mice and mice vaccinated once with PE(ΔIII)/E6 and/or PE(ΔIII)/E7 protein vaccines had tumorigenesis within 14 days after TC-1 tumor challenge (Figure **5C**), whereas 20%, 60%, and 100% of the mice vaccinated twice with PE(ΔIII)/E6, PE(ΔIII)/E7, and PE(ΔIII)/E6+PE(ΔIII)/E7 protein vaccines, respectively, were tumor-free 60 days after TC-1 tumor challenge (Figure **5D**).

Comparing the vaccine potency in the mice given three vaccinations of each respective protein vaccine through the therapeutic subcutaneous tumor injection model, the mice vaccinated with PE(ΔIII)/E6, PE(ΔIII)/E7, and PE(ΔIII)/E6+PE(ΔIII)/E7 fusion protein vaccines had small tumor volumes than those vaccinated with E6+E7 (*p*<0.001, one-way ANOVA) (Figure **5E**). The mice vaccinated with the PE(ΔIII)/E6+PE(ΔIII)/E7 fusion protein vaccines had the smallest tumor volumes 55 days after TC-1 tumor challenge (*p*<0.001, one-way ANOVA).

These results indicated that a higher number of vaccinations of fusion protein vaccines could generate more potent immune responses and anti-tumor effects. The protein vaccines targeting two tumor antigens generated better anti-tumor effects than those targeting single specific tumor antigens.

## Discussion

Protein vaccines represent another successful example of using the translocation domains of a bacterial toxin for the development of cancer vaccines and immunotherapy. The translocation domains of a bacterial toxin linked to the tumor antigen E6 in a protein format generated potent E6-specific immunity and E6-specific anti-tumor effects in this study. A previous study demonstrated that the translocation domain of *Pseudomonas aeruginosa* exotoxin A with HPV type 16 E7 tumor antigen, regardless of in a DNA or protein vaccine, enhanced vaccine potency. This suggests that the bacterial domain may serve as a useful tool for introducing exogenous protein into the cytosol. Fominaya et al. demonstrated that the truncated forms of the chimeric protein of a bacterial toxin linked with an antigen did not facilitate efficient protein or DNA transfer when lacking KDEL, the signaling transducer of the translocation domain [31]. However, domain II of the exotoxin without KDEL, when linked to the tumor antigen, generated potent immunity in the DNA vaccine.

The same strategy to enhance antigen-specific immunity can be applied to different tumor antigens. The PE(ΔIII)-E6-KDEL and PE(ΔIII)-E7-KDEL fusion protein vaccines generated potent E6- and E7-specific immunity in the present study. The number of E6-specific, IFN-γ-secreting CD4^+^ T cells in the PE(ΔIII)/E6 (219.5±18.9) group was significantly higher than that of the E6 (12.0±2.8) group (Figure **2A**). The number of E6-specific, IFN-γ-secreting CD8^+^ T cells in the PE(ΔIII)/E6 group (258.0±18.2) was also significantly higher than that of the E6 (15.0±2.8) group (Figure **2B**). In addition, there were more E7-specific, IFN-γ-secreting CD4^+^ T cells (247.5±18.2) (Figure **2C**) and CD8^+^ T cells (588.5±16.8) (Figure **2D**) in the PE(ΔIII)/E7 group than in the E7 group (14.5±2.1 for CD4^+^ T cells; 12.5±2.1 for CD8^+^ T cells). Moreover, E6- and E7-specific Abs titers were higher in the PE(ΔIII)/E6 (Figure **2E**) and PE(ΔIII)/E7) (Figure **2F**) groups than those in the E6 and E7 groups, respectively. Further studies investigating whether translocation domains of a bacterial toxin can be linked to other tumor antigens against other types of cancer are warranted.

A combination of fusion protein vaccines with PE(ΔIII)/E6 and PE(ΔIII)/E7 generated more potent immunotherapeutic effects than PE(ΔIII)/E6 or PE(ΔIII)/E7 fusion proteins alone. Walter et al. reported that the cancer vaccine MA901, consisting of multiple tumor-associated peptides combined with cyclophosphamide, generated immune responses to multiple tumor-associated peptides and provided longer overall survival [32]. A previous study also demonstrated that heat shock protein 60 co-linked to HPV16 E6 and E7 tumor antigens generated more potent immunotherapeutic effects than E6 or E7 tumor antigens alone in the DNA format [24]. The present study again demonstrated that PE(ΔIII)/E6 combined with PE(ΔIII)/E7 fusion protein vaccines generated more potent immune responses (Figure **2**) and anti-tumor effects (Figures **4** and **5**) than the respective fusion protein vaccines alone. This suggests that HPV E6 and E7 can be utilized together as target antigens in cancer vaccines and immunotherapy.

The mechanisms of the anti-tumor effects using the translocation domains of a bacterial toxin are via similar immuno-effector cells regardless of the targeted antigen. The bacterial toxin (PE(ΔIII)) strategy has been previously tested in a mammalian expressing DNA vector (pcDNA3) [33], and the CD8^+^ cytotoxic T lymphocytes were found to be the only essential immuno-effector cells for the anti-tumor mechanisms of the PE(ΔIII)/E7 naked DNA vaccine. However, the immuno-effector cells in the anti-tumor mechanism of the PE(ΔIII)/E7 fusion protein vaccine include CD4^+^ helper T lymphocytes, CD8^+^ cytotoxic T lymphocytes, and NK cells [15]. Therefore, different types of vaccines encoding the same construct seem to be able to activate different subsets of effector cells in the vaccinated host leading to different immunological or anti-tumor mechanisms.

The anti-tumor mechanisms of PE(ΔIII)/E7, PE(ΔIII)/E6, or PE(ΔIII)/E6+PE(ΔIII)/E7 fusion protein vaccines were the same in this study (Figure 3), even though the anti-tumor effects of PE(ΔIII)/E6+PE(ΔIII)/E7 fusion protein vaccines were stronger than those of the respective fusion protein vaccines alone. Thus, the same strategy to enhance the potency of cancer vaccines linked to different tumor antigens may activate similar subsets of effector cells and have same anti-tumor mechanisms.

The PE(ΔIII)/E7 fusion protein generated a stronger anti-tumor effect than the PE(ΔIII)/E6 fusion protein. Most previous research on HPV has focused on E7 [34]. Since E6 is another important target for potential vaccines to control HPV-associated lesions, it is crucial to develop vaccines targeting E6. The E6-specific immune responses generated by the E6-specific vaccine were weaker than those of the E7-specific vaccine in the same HSP60 DNA [24] and PE(ΔIII) protein formats in this survey. Furthermore, the anti-tumor effects of HSP60/E6 DNA and PE(ΔIII)/E6 fusion protein vaccines were weaker than those of the HSP60/E7 DNA and PE(ΔIII)/E7 fusion protein vaccines. The size of E6 (about 150 aa) may be the reason why it is a weaker antigen than E7 (about 100 aa). Nevertheless the vaccines targeting both E6 and E7 tumor antigens had a better anti-tumor effect than those targeting only E6 or only E7 tumor antigens, regardless of the DNA or protein format. The combination of E6 and E7 as target tumor antigens is important in aiding the future development of HPV vaccines.

Protein vaccines have some advantages over naked DNA vaccines. First, protein vaccines can induce various kinds of antigen-specific effector T cells such as CD4^+^ helper and CD8+ cytotoxic lymphocytes, as shown in this study. However, naked DNA vaccines only induce CD8^+^ cytotoxic lymphocytes [17,35]. Protein vaccines are also more convenient to prepare using the present techniques compared to naked DNA vaccines. Furthermore, naked DNA vaccines carry the risk of DNA integration into the host genome, although it is estimated that the frequency of such integration is low [36]. Nevertheless, such concerns of DNA integration are avoided with protein vaccines. The two PE(ΔIII)/E6 and PE(ΔIII)/E7 fusion protein vaccines can be used in future human clinical trials for the prevention of HPV infection and as therapy for HPV-related cancers.

In summary, targeting two tumor antigens, HPV E6 and E7, together enhanced the potency of protein fusion vaccines. The mechanisms of the fusion protein vaccines were found to be through effector cells including antigen-specific CD4^+^ and CD8^+^ T cells, and NK cells. Because most cervical cancers express HPV E6 and E7, the proposed vaccine has the potential for use in the prevention and treatment of HPV-associated tumors. The strategy of using the translocation properties of bacterial toxins and two target antigens may hold promise in the future development of vaccines for the control of cancers and infectious diseases.
